# Effect of acute mid-intensity treadmill exercise on the androgen hormone level and uncoupling protein-1 expression in brown fat tissue of mouse

**DOI:** 10.20463/jenb.2018.0003

**Published:** 2018-03-31

**Authors:** Nahyun Kim, Jisu Kim, Choongsung Yoo, Kiwon Lim, Takayuki Akimoto, Jonghoon Park

**Affiliations:** 1. Department of Physical Education, Korea University, Seoul Republic of Korea; 2. Laboratory of Exercise Nutrition, Department of Physical Education, Konkuk University, Seoul Republic of Korea; 3. Laboratory of Muscle Biology, Faculty of Sport Sciences, Waseda University, Tokorozawa Republic of Korea

**Keywords:** Exercise, Brown adipose tissue, Androgen hormone, Thermogenesis, UCP-1

## Abstract

**[Purpose]:**

Brown adipose tissue (BAT) plays an important role in metabolizing different substances, including androgens. The aim of this study was to determine whether a single bout of aerobic exercise would increase the androgen hormone concentration in mouse BAT and whether its increase was associated with uncoupling protein-1 (UCP-1), protein kinase A (PKA)-related mechanism in BAT.

**[Methods]:**

Twenty, 9-week-old ICR adult male micewere randomly divided into three groups: Control (*n*=6, CON), Exercise (*n*=7, EX), and Exercise + SRD5A1A2 inhibitor (*n*=7, EXIN). SRD5A1A2 is an enzyme needed when free testosterone is metabolized to dihydrotestosterone (DHT). SRD5A1A2 was administered intraperitoneally in the EXIN group, while the CON and EX groups were treated with the vehicle only. One hour later, exercise was performed at 60–70% ${\dot{V}O}_{2max}$ for 30minutes. The levels of testosterone and DHT in BAT were determined by ELISA, and UCP-1 mRNA level was examined by RT-PCR. UCP-1 and PKA protein levels were determined by western blotting.

**[Results]:**

After a single period of exercise, testosterone and DHT concentrations in BAT were significantly higher in EX than those in CON, and lower in EXIN than those in EX. The ratio of phosphorylated PKA to total PKA in BAT was significantly higher in EX than that in CON, and lower in EXIN than that in EX. UCP-1 levels in BAT were not different in the three groups.

**[Conclusion]:**

Aerobic exercise increased bioactive androgen hormone levels in BAT in association with the increase in phosphorylated PKA levels. In contrast, 30minutes of treadmill exercise did not affect UCP-1 expression.

## INTRODUCTION

The global prevalence of obesity continues to increase. Obesity, which is defined as the excessive accumulation of fat in the human body, causes various complications and hasextremely negative effects on human health^1^. According to the 2015 Korea National Health and Nutrition Examination Survey(KNHANES)^2^, the prevalence of obesity in Korean adults increased from 26.0% in 1998 to 36.6% in 2012, and men showed a particularly dramatic rise from 25.1% to 39.7%. Obesity causes various health problems both independently and in association with other diseases^3^. It is a direct cause of type 2 diabetes, cardiovascular diseases, and metabolic diseases, such as hypertension^3,4^.

The biggest cause of obesity is the energy imbalance created due to an increased calorie intake or reduced energy consumption. Decreased energy consumption may be due to decreased levels of sex hormones and reduced activity of brown adipose tissue (BAT).Adipose tissues in the human body are generally categorized into white adipose tissue (WAT), which stores energy in the form of neutral fat, and BAT, which is responsible for energy consumption through thermogenesis. There is relatively little BAT compared to WAT. BAT appears brown because it contains mitochondria that are enriched in iron. Uncoupling protein-1 (UCP-1)is specifically expressed and localized on the inner wall of the mitochondria present in BAT. UCP-1 induces non-shivering thermogenesisthat results in increased energy consumption^5,6^.The UCP-1-mediated production of heat is stimulated by the sympathetic nerve systemunder the influence of cyclic adenosine monophosphate (cAMP) and protein kinase A (PKA)^7^.BAT activity decreases the level of body fats, including neutral fats due to increased energy consumption^8-10^.

Recent studies reported that the suppression of the expression of sex hormones in mice increases UCP-1 expression,which induces heat production in BAT and the consequent decline in energy consumption^11,12^. In knockout models of androgen receptors—to which male hormones, such as testosterone and dihydrotestosterone (DHT),naturally bind—energy consumption declined due to the fall of UCP-1 expression in BAT and the mice became obese^11,12^.Consistent with this, the fat tissue content increased in knockout mice, whose receptors of the female hormone,estrogen, are completely knocked out over the course of their growth^13^. The collective results indicate that decreased levels of sex hormones are the major cause of obesity.

Sex hormones were thought to be synthesized and secreted by the endocrine glands of the ovary and testis. However, the results from recent studies indicate that sex hormones may be produced and secreted by peripheral tissues other than the gonads, such as the brain and skeletal muscles^14^. Dehydroepiandrosteron (DHEA) is a precursor of sex hormones. DHEA is metabolized to testosteroneby the activity of 17β-hydroxysteroid dehydrogenase (17β-HSD). DHT is generated from testosterone due to the increased expression of 5α-reductase (a representative example is SRD5A1), and the metabolism transforms into a pathway linked to androgen receptors ^15^. Androgen receptors are also present in BAT, which is responsible for energy consumption through heat generation. Sex hormones influence BAT through the sympathetic nervous system^16^. A significant decrease inUCP-1levels was described in mice whose androgen receptors were inhibited^11,17^, and it is likely that the androgen hormonehas a direct effect on UCP-1 expression.

In a study by Aizawa et al. (2010), 10-week-old male and female adult mice engaged in a single bout of mid-intensity treadmill exercise, followed by analysis of the levels of sex hormones and enzymes related to sex hormone synthesis in the gastrocnemius. Increased levels of testosterone and DHTandrogen hormone were observed in the muscles instead of the endocrine glands of the testis. In particular, levels of 5α-reductase type 1, which is necessary for the synthesis of DHT from testosterone, was significantly increased^18^. When the mice were subjected to a long-term treadmill endurance exercise regime, the expression of the androgen hormone synthase 5α-reductase (SRD5A1 isoform) and DHT increased in skeletal muscles^19^. These results provided the first indication of the possibility of exercise-induced androgen steroidogenesis in local tissues. However, little is known concerning exercise associated synthesis of sex hormone in local BAT.

Matteis et al. (2013) observed the decreased size of adipose droplets in BAT and increased expression of UCP-1 in mice engaged in mid-intensity endurance exercise compared to those in the controlmice^20^. Xu et al. (2011) reported that mid-intensity endurance exercise increased the expression of UCP-1 in BAT in obese mice conditioned by an 8-week hypercaloric diet regime ^21^. No study has directly examined the effect of exercise associated increased androgen hormone levels in BAT on UCP-1 expression. In this study we hypothesized that mid-intensity treadmill exercise activates the sex hormone production pathway in local BAT and that the expression of UCP-1 will be increased through protein kinase A (PKA) activity. To prove this hypothesis, ICR male mice were subjected to a single bout of mid-intensity treadmill exercise. We assessed the changes in androgen hormone concentration in BAT and analyzed the effect of BAT androgen hormone on PKA and UCP-1 expression.

## METHODS

### Study subjects

Twenty 9-week old mature male ICR mice were fed experimental animal feed and acclimatized to the experimental environment for a week, prior to experimentation. Sex hormones are unstable during the growth and aging periods. Thus, we used mature (9-week old) mice.

### Study design

**Fig.1.**
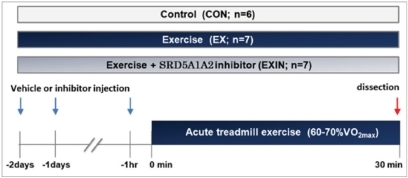
Experimental design

After the preliminary acclimatization, the mice were randomly allocated into control (CON), exercise(EX), and exercise + DHT inhibitor (EXIN) groups(Figure 1). Previously prepared inhibitor of the SRD5A1 and SRD5A2 isoforms of 5α-reductase (Avodart; dutasteride 2mg/kg) diluted in fat-soluble oil was injected into the percutaneous fat in a shoulder blade of the EXIN group 2 days, 1 day, and 1 hour prior to the mid-intensity exercise^22^. The CON and EX groups were administered the same fat soluble vehicle. Autopsy was performed in a consecutive manner 1 hour after the last administration and immediately after the 30-minute 60-70% ${\dot{V}O}_{2max}$ mid-intensity treadmill exercise. Experimental mice were fasted for 12 hours (Figure 1). They were anesthetized with etherimmediately after the experiment, and 10ml of blood was sampled from the main artery. Interscapular-BAT (iBAT) was taken from the dissected shoulder blade area. It was weighted and stored in a freezer at –80℃ untilanalyzed.

### Exercise program

The mice were induced to exercise for 30 minutes at 8 m/min for 5 minutes; 13 m/min for the next 5 minutes; and 18 m/min for the remaining 20 minutes on a special gas chamber treadmill designed to rodents energy metabolism (Figure 8). When mice arbitrarily paused during the exercise, they were induced to continue the workout using 30 volts of electricity delivered through the bottom of the treadmill belt and by brushing of the tail.

### Analysis

#### Protein

Protein analysis was via western blot. PKA, phosphorylated PKA(p-PKA), and UCP-1 were measured for gene expression associated with heat production. β-tubulin was measured as an internal control protein. Tissues were dissolved in lysis buffer (50mM Tris- HCL pH 7.5, 100mM NaCL, 1mM EDTA, 2% sodium dodecyl sulfate [SDS], 0.1mM phenylmethylsulfonyl fluoride, and 2μg/ml leupeptin)at 4℃ for 1 hour. Protein was extracted by centrifugation at 4℃ and 14,000rpm for 30 minutes. The extracted protein was quantified by the Bradford method using a kit (Bio-Rad, Richmond, CA, USA). A designated quantity of total protein was mixed with sample buffer containing SDS andβ-mercaptoethanol, boiled at 95℃ for 5 minutes, and processed by 12% sodium dodecyl sulfate-polyacrylamide gel electrophoresis. The resolved proteins were transferred to a nitrocellulose membrane using wet transfer. The membrane was incubated with primary antibodies at 4℃ overnight, washed, incubated with secondary antibodies for 1 hour, and was washed. After the final wash, the membrane was exposed to ECL reagent (Amersham Life Science, Little Chalfont, UK) and autoradiography was performed. The following antibodies were used:UCP-1 (1:1000, sc-6529; Santa Cruz Biotechnology, Dallas, TX, USA);PKA C-alpha (1:1000, 4782; Cell Signaling Technology, Danvers, MA, USA);phosphorylated-PKA C (Thr197) (1:1000, 4781; Cell Signaling Technology);and β-tubulin (1:1000, 2128; Cell Signaling Technology).

#### mRNA

The messenger RNA level of UCP-1 was measured as a data subset of protein analysis. The isolated muscles and adipocytes were homogenized with TRIzolreagent (Invitrogen, Carlsbad, CA, USA). Chloroform was added (200μl) to each1ml tube. The tubes were shaken to mix the solution, incubated at room temperature for 5 minutes, and centrifuged at 4℃ for 10 minutes (12500 x g) to isolate RNA. Total RNA of each group was quantified using a SmartspecTM Plus spectrophotometer (Bio-Rad). To observe the reaction of the RNA reverse transcription, diethyl pyrocarbonate, 10mMdNTI, andoligo (dT) were added to 5 μg of total RNA, and the solution was incubated in a T3000 thermocycler PCR machine (Biometra, Göttingen; Germany) and cDNA was obtained. The RT-PCR products of the genes expressed from the muscle and adipocytes were resolved by 2% agarose gelelectrophoresis at 100 V. The image of the gel was captured using an ultraviolet transilluminator, and the concentration of the mRNA expression was measured using Gel Doc 2000 device (Bio-Rad). The forward and reverse primers for UCP- 1were 5′-AAG AGG AAG GGA CGC TCA C-3′ and 5′-TCG GAA GTT GTC GGG TTG-3′, respectively.

#### Enzyme-linked immunoassay

Commercial ELISA kits were used to quantify testosterone and DHT (both from IBL International GMBH, Flugha–fenstrasse, Hamburg, Germany). The assays were performed, as described by the manufacturer. Levels of testosterone and DHT were measured at the manufacturer recommended absorption wavelengths using a microplate reader.

#### Statistical analyses

Data were analyzed using SPSS version 23.0 software (IBM, Armonk, NY, USA). The calculated mean (M) ±standard error of the mean (S.E.M) values provided descriptive statistics of all dependent variables. The significance level was <5%. One-way ANOVA was conducted to test the average difference of the dependent variables of the two groups (e.g. physical composition and biochemical variables of BAT).When a significant F-value was observed, a post-hoc test (least significant difference, LSD) was performed to test significant differences between the groups. Pearson correlation test was performed to test the correlation between androgen and PKA and UCP-1 of BAT.

## RESULTS

This study investigated the effect of aerobic exercise on the level of androgen, UCP-1 gene expression, and energy consumption in BAT. ICR mice were induced to perform one session of mid-intensity exercise for 30 minutes on a treadmill. Testosterone, DHT, UCP-1 mRNA expression, and protein levels of UCP-1 and PKA were measured in BAT.

**Fig.2.**
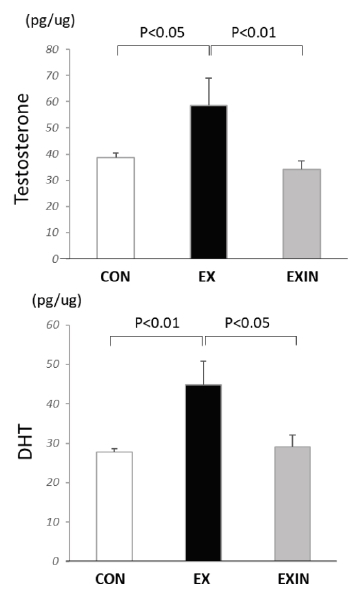
Change in androgen hormone levels in brown fat DHT, dihydrotestosterone; CON, control; EX, exercise; EXIN, exercise+DHT inhibitor. Data are expressed as mean ± SE.

After treadmill exercise, the concentration of testosterone and DHT in BAT significantly increased in the EX group compared to that in the CON group. The levels of DHT and testosterone were significantly decreased in the EXIN group. In the mice in the EXIN group, DHT production was inhibited prior to exercise compared to that in the exercise-only EX group of mice (Figure 2).

**Fig.3.**
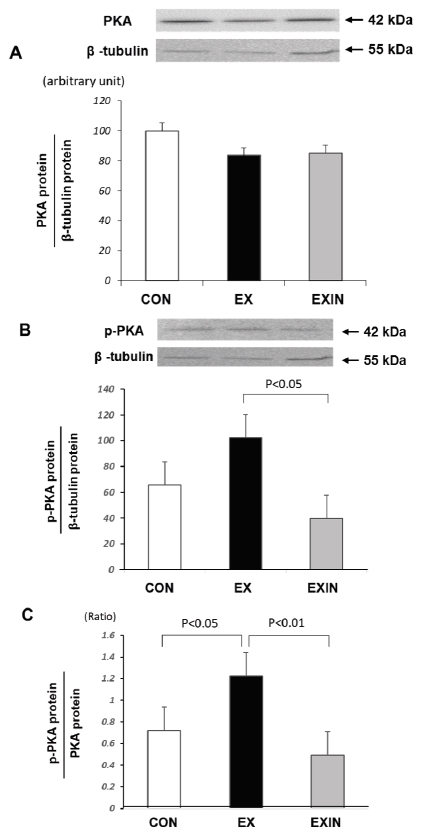
Change in PKA protein levels in brown fat PKA, protein kinase A; CON, control; EX, exercise; EXIN, exercise+DHT inhibitor. Data are expressed as mean ± SE.

The concentration of total PKA was not significantly different between the CON, EX, and EXIN groups (Figure 3A). The p-PKA concentration was not significantly different between the EX and CON groups, but was significantly higher in the EX group compared to that in the EXIN group (Figure 3B). The ratio of p-PKA to total PKA was significantly higher in the EX group compared to that in the CON groups and to that in the EXIN group, in which DHT was deactivated prior to exercise (Figure 3C).

**Fig.4.**
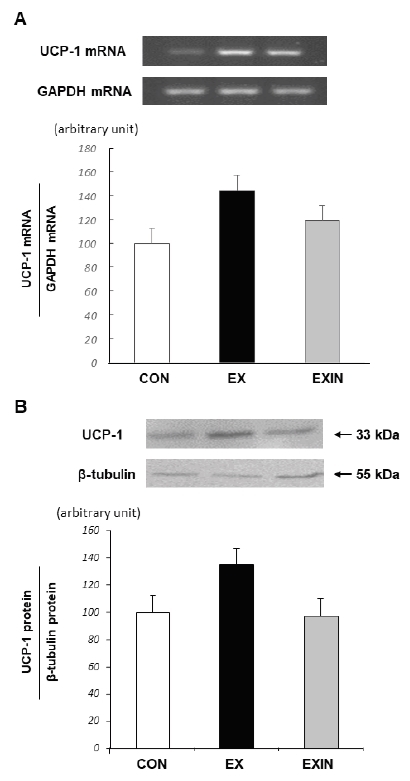
Change in UCP-1 mRNA (A) and UCP-1 protein (B) expression in brown fatCON, control; EX, exercise; EXIN, exercise+DHT inhibitor. Data are expressed as mean ± SE.

The expression of UCP-1 mRNA was not significantly different between the EX and CON groups (*p*=0.156) or between the EX and EXIN groups (*p*=0.420) (Figure 4A). The expression of UCP-1 was not significantly different between the EX and CON groups (*p*=0.165) or between the EX and EXIN groups (*p*=0.138) (Figure 4B).

**Fig.5.**
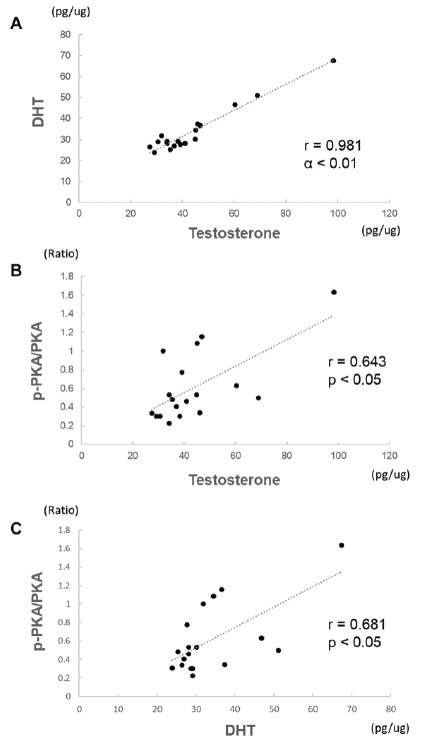
Association between DHT and testosterone (A), between p-PKA/PKA and testosterone (B), and between p-PKA/PKA and DHT (C)

DHT and testosterone concentrations were significantly correlated in Pearson correlation analysis (*r*=0.981, *p*<0.01) (Figure 5A). PKA activity was significantly associated with the concentration of testosterone (*r*=0.643, *p*<0.05) (Figure 5B) and DHT(*r*=0.681, *p*<0.05) (Figure 5C).

## DISCUSSION

The single session of 30minutesof mid-intensity treadmill exercise increased the levels of testosterone and DHT in BAT. The ratio of p-PKA to total PKA was increased by the exercise. However, the levels of androgen hormone and PKA were inhibited in the EXIN group of mice. On the other hand, the exercise did not have any effect on increase in UCP-1 expression.

Aizawa et al. (2010) reported that a 30-minute treadmill exercise increased testosterone and DHT levels in the muscles of 10-week-old female and male mice. Further, the levels of 5α-reductase, a synthase biosynthesizing DHT from testosterone, and androgen hormone were increased following exercise^18^. These findings suggest the possibility of exercise-related synthesis of sex hormones. In our study, therefore, the expression of 5α-reductase in BAT may have increased, or androgen hormone may have been synthesized in muscles and other tissues as a result of the exercise. These are the likely causes of our finding of increased androgen hormone levels in BAT after exercise.

PKA phosphorylation was also increased after the exercise. Both androgen hormone and PKA activity were reduced when inhibitors of the SRD5A1 and SRD5A2 isoforms of 5α-reductase were administered. The results suggest that the exercise-related increase in androgen hormonelevels may influence PKA activity. PKA is activated by phosphorylation, and the degree of PKA activity is determined by its ratio of phosphorylation. The activated protein influences UCP-1 expression by stimulating peroxisome proliferator-activated receptor gamma coactivator 1-alpha (PGC1-α) in adipose issues^7^. Kana et al. (2014) observed that voluntary 8-week wheel running exercise by obese mice increased both cAMP, which influences PKA activity in BAT, and PKA activity^23^. Martínez et al. (2014) reported that the androgen hormone, estrogen, stimulates beta-3 adrenergic receptor (β3-AR) in BAT by stimulating sympathetic nerves, and controls thermogenesis through the PKA-PGC1-α pathway^24^. Estrogenis one of the sex hormones synthesized from testosterone^15^. In our study, the exercise-related increase intestosterone levels in BAT may have increased estrogen levels, ultimately increasing PKA phosphorylation.

Lorna et al. (2016) found that PKA activity influences the increase in UCP-1^15^.Other studies reported that increased levels of sex hormones influence the expression of UCP-1^11,12^. Conversely, in the present study, exercise influenced the increase inandrogen hormonelevels in BAT and PKA activity, but did not have any effect on UCP- 1 expression. Shen et al. (2016) reported increased UCP-1 mRNA expression in 10-week-old mice after a single 120-minute session of swimming exercise^25^. Jalil et al. (2016) reported that an 8-week resistance training regimen increased the expression of UCP-1 mRNA in mature male mice^26^. Kana et al. (2014) observed that low-intensity voluntary wheel running exercise for 8 weeks increased UCP-1 expression in WAT but not that in BAT in obese mice^21^. It is possible that the shorter duration and lower intensity of our exercise protocol might have been insufficient to have any tangible effect on UCP-1 expression. Koh et al. (2007) reported thata single session of exercise increased the expression of AMP-activated protein kinase, a downstream target of PKA, in adipocytes^27^.Therefore, it would be prudent to investigate the pathway from PKA to UCP-1. In our study, the single session of exercise at 60- 70% ${\dot{V}O}_{2max}$ intensity for30 minutes focusing on local androgen concentration levels was done using as previously described^18^.

There are several limitations of our study. First, the number of mice used for the experiment satisfied the statistical test level, but six or seven mice is a small sample size. Second, it is quite likely that the exercise intensity and duration may have been insufficient to increase UCP-1 levels. In a future study, it will be necessary to modify the exercise intensity and duration, with a prolonged exercise intervention. An exercise protocol different from that focused on increasing androgen hormone levels should be used as well.

In conclusion, a single 30-minute session of mid-intensity treadmill exercise increased the levels of testosterone and DHT in BAT. This exercise protocol did not affect UCP-1. It is possible that the exercise-related increase in DHT levels in BAT influenced the increased PKA activity. Hormone replacement therapies are being intensively studied as a means of solving various metabolic disorders, including obesity, which can affect men and women as sex hormone production declines with age^28,29^. However, these therapies have considerable side effects. The increased androgen hormone level in BAT after a single bout of exercise in our study provides evidence that physical exercise could be an alternative to hormone therapies.
